# Die Medizininformatik-Initiative als Wegbereiter für die datengetriebene Gesundheitsforschung in Deutschland

**DOI:** 10.1007/s00103-024-03885-7

**Published:** 2024-06-11

**Authors:** Martin Sedlmayr, Sebastian Claudius Semler

**Affiliations:** 1 Institut für Medizinische Informatik und Biometrie & Zentrum für Medizinische Informatik, Medizinische Fakultät Carl Gustav Carus, TU Dresden, Fetscherstraße 74, 01309 Dresden, Deutschland; 2 TMF – Technologie- und Methodenplattform für die vernetzte medizinische Forschung e. V., Charlottenstraße 42, 10117 Berlin, Deutschland

Die zunehmende Digitalisierung des Gesundheitswesens eröffnet neue Möglichkeiten für die Forschung und die Versorgung von Patientinnen und Patienten. Um diese Möglichkeiten zu nutzen, hat das Bundesministerium für Bildung und Forschung (BMBF) 2016 die Medizininformatik-Initiative (MII) ins Leben gerufen.

Die MII zielt darauf ab, als Grundlage für die Chancen der Digitalisierung Infrastrukturen aufzubauen, um Datenverfügbarkeit und Datenzugang für die medizinische Forschung zu verbessern. Hierzu gehört insbesondere, Daten aus der Routineversorgung für die medizinische Forschung zugänglich zu machen. Für diese Aufgabe werden nach einem harmonisierten Vorgehensmodell Datenintegrationszentren (DIZ) an allen Universitätskliniken und ersten außeruniversitären Partnerinstitutionen aufgebaut und miteinander bundesweit vernetzt. Die DIZ und das zentrale Deutsche Forschungsdatenportal für Gesundheit (FDPG) bilden zusammen eine dezentral föderierte Digitalinfrastruktur für die medizinische Forschung in Deutschland.

Dabei befasst sich die MII nicht allein mit technischen Entwicklungen. Eine einheitliche Rechtsgrundlage und ein von allen Universitätskliniken unterzeichnetes Vertragswerk sind wichtige Voraussetzungen für eine bundesweite Datennutzung. Ein bedeutender Schritt hierfür war die Einführung einer mit allen Datenschutzaufsichtsbehörden der Länder und des Bundes sowie den Ethikkommissionen abgestimmten „breiten Einwilligungserklärung“ (Broad Consent), welche die ethisch und rechtlich zulässige Nutzung von Patientendaten, Kassendaten und Biomaterialien ermöglicht. Für die bundesweite Datennutzung ist zudem die notwendige Einigung auf einheitliche Datennutzungsprozesse und Datenstandards (wie FHIR^1^) erfolgt. Dadurch entstand ein nationales Netzwerk für die Sekundärnutzung von Versorgungsdaten.

Die Initiative führte zur Besetzung von über 50 neuen Professuren und der Etablierung von mehr als 25 Nachwuchsgruppen. In der aktuellen Förderphase seit 2023 werden 11 übergreifende Use Cases gefördert, welche auch eine zunehmende Versorgungsrelevanz besitzen.

Die MII arbeitet nicht isoliert, sondern in einem größeren Kontext. In den letzten Jahren wurden viele Netzwerke für vernetzte medizinische Forschung geschaffen, wie z. B. das Netzwerk Universitätsmedizin (NUM), die Nationale Forschungsdateninfrastruktur (NFDI) und genomDE. Diese Netzwerke streben zunehmend nach Synergien und Interoperabilität, um einen starken Forschungsraum in Deutschland zu schaffen. Aktuelle Gesetzgebung im nationalen wie europäischen Rahmen, insbesondere das deutsche Gesundheitsdatennutzungsgesetz (GDNG) und die kommende europäische Verordnung zum Europäischen Gesundheitsdatenraum (EHDS), bietet der MII und ihren kooperierenden Netzwerken perspektivisch deutlich erweiterte Möglichkeiten, Daten zu gemeinwohlorientierten Zwecken in der digitalen Medizin und zur Verbesserung der Patientenversorgung und Patientensicherheit zu nutzen. Mit den aus der MII resultierenden Infrastrukturen und Verfahrensweisen hat sich Deutschland bereits zu einem guten Teil für einen Anschluss an die harmonisierte Sekundärnutzung von Gesundheitsdaten im EHDS fit gemacht.

In diesem Themenheft werden die Ergebnisse der MII präsentiert sowie einige der aktuellen Use Cases vorgestellt. Darüber hinaus wird ein Ausblick auf ergänzende und vernetzende Maßnahmen gegeben.

Das Themenheft beginnt mit einem Überblick von *Semler et al.* über die Entwicklung, Struktur und Governance der MII, die 4 Konsortien der MII (DIFUTURE, HiGHmed, MIRACUM, SMITH), die Koordinationsstrukturen und die sie tragenden Verbände (TMF^2^, MFT^3^, VUD^4^) sowie die weiteren Fördermodule und Kooperationen. Anschließend wird ein Überblick über wichtige Ergebnisse der MII gegeben wie den Broad Consent als Grundlage der Datennutzung, den Kerndatensatz in FHIR und die harmonisierten Regelungen und Prozesse des Data Sharing. Das FDPG spielt dabei eine zentrale Rolle für die Nutzung durch Forschende.

Das Herzstück der MII bilden die DIZ, die an den Unikliniken etabliert wurden. *Albashiti et al.* beschreiben die notwendigen technischen, organisatorischen und ethisch-datenschutzrechtlichen Rahmenbedingungen, die es ermöglichen, klinische Routinedaten nicht nur lokal, sondern auch standortübergreifend für Forschungszwecke nutzbar zu machen.

Im folgenden Abschnitt wird exemplarisch die Arbeit dreier – von insgesamt 5 – Arbeitsgruppen der MII vorgestellt, die den nationalen Rahmen zur Datennutzung geschaffen haben.

Die Arbeitsgruppe (AG) Consent hat vor allem den modularen Broad Consent entwickelt und mit den Ethikkommissionen und Datenschutzbehörden abgestimmt, um die Nutzung von Patientendaten, Kassendaten und Bioproben zu ermöglichen. *Zenker et al.* berichten in ihrem Artikel über die Erfahrungen bei der Einführung des Broad Consent an den Standorten.

Die AG Data Sharing hat Nutzungsbedingungen, rechtliche Regelungen und Datenzugriffsprozesse entwickelt, die von den DIZ und den Use and Access Committees an allen Unikliniken einheitlich umgesetzt werden. *Kirsten et al.* beschreiben zudem Services, die Forschenden einen standardisierten Datenzugang ermöglichen.

Die AG Interoperabilität hat insbesondere den modularen, standardisierten Kerndatensatz der MII entwickelt und dient als Plattform für die Abstimmung übergreifender Vorgehensweisen, Datenstrukturen und Schnittstellen zwischen den DIZ sowie nationalen und internationalen Interoperabilitätsgremien. *Ammon et al.* stellen in ihrem Artikel die Arbeitsweise der AG vor, insbesondere den Entwicklungsprozess und Release-Wechsel sowie wesentliche Ergebnisse wie die Kerndatensatzmodule, Terminologiedienste und Metadaten, die zentrale und dezentrale Auswertungen ermöglichen.

Im dritten Abschnitt werden exemplarisch 3 von insgesamt 11 geförderten Use Cases vorgestellt.

*Metzger und Börries* beschreiben das Projekt PM4Onco – Personalisierte Medizin für die Onkologie. Dieses Projekt konzentriert sich auf die Etablierung und Harmonisierung molekularer Tumorboards, bindet Krebsregister ein und schafft Synergien mit Netzwerken wie dem Deutschen Konsortium für Translationale Krebsforschung (DKTK).

*Löffler et al.* stellen die prospektive, interventionelle Studie in der Krankenversorgung zur Verbesserung der Arzneimitteltherapiesicherheit INTERPOLAR vor. Dabei wird ein Tool evaluiert, das Stationsapothekerinnen und -apotheker unterstützt, indem es sie auf Patientinnen und Patienten mit potenziell kritischen Medikationsverordnungen aufmerksam macht.

Der dritte Use Case im Artikel von *Marschollek et al.* befasst sich mit der automatisierten Überwachung und Risikovorhersage zur risikostratifizierten Infektionskontrolle und -prävention (RISK PRINCIPE). Das Ziel ist es, Präventionspotenziale und Risikofaktoren bei nosokomialen Infektionen zu identifizieren, um gefährdete Patientinnen und Patienten zu erkennen, die von spezifischen oder zusätzlichen Interventionen profitieren würden.

Der vierte Abschnitt beschäftigt sich mit begleitenden Maßnahmen zur Stärkung der Medizininformatik.

Der Artikel von *Knaup-Gregori et al.* fasst die Vielzahl an Aktivitäten zusammen, die in den Konsortien der MII zur Aus- und Weiterbildung entwickelt wurden. Dabei wurden nicht nur über 50 neue Professuren und 10 neue Masterstudiengänge geschaffen, sondern auch vorwiegend digitale Lernangebote für Forschende wie Clinician Scientists erstellt, um ihnen die Nutzung der entwickelten Werkzeuge zu erleichtern.

Während sich die MII zunächst auf die Universitätsmedizin konzentrierte, sollen die „Digitalen Fortschrittshubs Gesundheit“ die Erweiterbarkeit der Konzepte und Lösungen der MII für eine Verbesserung der regionalen Gesundheitsversorgung und -forschung pilotieren. *Krefting et al.* stellen in ihrem Artikel die 6 geförderten Hubs vor und resümieren die besonderen Herausforderungen des nichtuniversitären Sektors.

Im letzten Artikel des Themenhefts geben *Waltemath et al.* einen Ausblick auf FAIRe Gesundheitsdaten, d. h. Daten die sich öffentlich leicht finden und wiederverwenden lassen, im nationalen und internationalen Datenraum. Dabei werden auch Potenziale der Anknüpfung an nationale Initiativen wie NFDI und internationale Netzwerke wie „Observational Health Data Sciences and Informatics“ (OHDSI) beschrieben.

Zusammenfassend hat die MII in den letzten Jahren bedeutende Fortschritte in der Schaffung einer vernetzten Infrastruktur für die medizinische Forschung in Deutschland gemacht. Dieses Themenheft bietet einen Überblick über die erzielten Ergebnisse, die in ihrer Wirkung auch über den Rahmen der MII hinausgehen. Erstmalig gibt es in Deutschland ein unter allen Unikliniken harmonisiertes Datenmodell, gemeinsame Nutzungsbedingungen und -verträge sowie ein zentrales Datenantragsportal. Zusätzlich werden im Themenheft Fortschritte in der Aus- und Weiterbildung sowie die Vernetzung mit anderen Fach-Communitys hervorgehoben. Diese Entwicklungen stärken die MII als zentrale Säule in der Forschung mit „Real World Data“ aus der Versorgung und fördern die Integration in nationale Gesundheitsforschungsstrukturen.

## Footnotes

1 Fast Healthcare Interoperability Resources

2 TMF: Technologie- und Methodenplattform für die vernetzte medizinische Forschung e. V.

3 MFT: Medizinischer Fakultätentag e. V.

4 Verband der Universitätsklinika Deutschlands e. V.

